# Minimally invasive epicardial left-ventricular lead implantation and simultaneous left atrial appendage closure

**DOI:** 10.3389/fcvm.2023.1129410

**Published:** 2023-03-10

**Authors:** Johannes Petersen, Yousuf Alassar, Yalin Yildirim, Tobias Tönnis, Hermann Reichenspurner, Simon Pecha

**Affiliations:** ^1^ Department of Cardiovascular Surgery, University Heart and Vascular Center Hamburg, Hamburg, Germany; ^2^ German Center for Cardiovascular Research (DZHK), Hamburg/Kiel/lübeck, Germany; ^3^ Department of Cardiology, University Heart and Vascular Center Hamburg, Hamburg, Germany

**Keywords:** minimally-invasive, totally thoracoscopic, epicardial lead placement, left atrial appendage closure, atrial fibrillation

## Abstract

**Background:**

Atrial fibrillation (AF) is common in patients with heart failure resulting in a high prevalence of AF in patients receiving Cardiac Resynchronization Therapy (CRT) implantation. In patients, unsuitable for transvenous left ventricular (LV)-lead implantation, epicardial LV-lead implantation represents a valuable alternative. Epicardial LV-lead placement can be achieved totally thoracoscopical or *via* minimally invasive left lateral thoracotomy. In patients with atrial fibrillation, concomitant left atrial appendage (LAA) clipping is feasible *via* the same access. Therefore, the aim of our study was the analysis of safety and efficacy of epicardial LV lead implantation and concomitant LAA clipping *via* minimally invasive left-lateral thoracotomy.

**Methods:**

Between December 2019 and March 2022, 8 patients received minimally invasive left atrial LV-lead implantation with concomitant LAA closure using the AtriClip. Transesophageal echocardiography (TEE) was performed to intraoperatively guide and control LAA closure.

**Results:**

Mean patients age was 64 ± 11.2 years, 67% were male patients. Minimally invasive left-lateral thoracotomy was used in 6 patients while a totally thoracoscopic approach was performed in 2 cases. Epicardial lead implantation was successfully performed in all patients with good pacing threshold (mean 0.8 ± 0.2 V) and sensing values (10.1 ± 2.3 mV). Posterolateral position of the LV lead was achieved in all patients. Furthermore, successful LAA closure was confirmed during TEE in all patients. No procedure-related complications occurred in any of the patients. Two patients additionally received simultaneous laser lead extraction during the same procedure. Complete lead extraction was achieved in both patients. All patients were extubated in the OR and had an uneventful postoperative course.

**Conclusion:**

Our study highlights a novel treatment approach for patients with atrial fibrillation and the necessity of epicardial LV leads. Placement of a posterolateral LV lead position with concomitant occlusion of the left atrial appendage *via* a minimally-invasive left-lateral thoracotomy or even a totally thoracoscopic approach is safe and feasible with superior cosmetic result and complete occlusion of the left atrial appendage.

## Introduction

Atrial fibrillation (AF) has a high prevalence in patients with heart-failure (1, 2) and consequently, in patients undergoing cardiac resynchronization therapy defibrillator (CRT-D) implantation (3).

In patients, not suitable for transvenous left ventricular (LV) lead implantation, epicardial lead implantation represents a valuable treatment alternative. Most recently, Burger et al. outlined excellent results of epicardial left-ventricular leads with comparable performance of transvenous leads (4). The advantage of epicardial leads is the selection of the ideal left-ventricular target area, independently from the anatomy of the coronary sinus vein. Furthermore, epicardial LV leads are essential treatment options in patients with systemic cardiac device related infections, which is a rare but severe complication of transvenous device therapy. In case of cardiac device infection, complete lead extraction is recommended in those patients (5). In such patients, especially with pacemaker-dependency, epicardial LV leads are helpful tools to ensure continuous stimulation until resolution of infection is achieved. Epicardial LV-lead placement can be achieved *via* totally thoracoscopic port-access or left-lateral mini-thoracotomy, allowing for a postero-lateral positioning of the LV-lead (6, 7). In patients with AF, concomitant surgical left-atrial appendage closure resulted in a significant stroke risk reduction (8) and therefore concomitant LAA closure is highly recommended in all AF patients undergoing any type of cardiac surgery. The AtriClip® (Fa. AtriCure, Cincinnati, Ohio) LAA exclusion system can be used in minimally- invasive surgery and allows for successful LAA closure through port-access (AtriClip PRO2®) or left-lateral minithoracotomy (AtriClip Pro®).

Therein, we report our initial experience with left-ventricular lead implantation and concomitant LAA closure *via* a thoracoscopic port-access- or left-lateral mini-thoracotomy**.**

## Methods

Between December 2019 and March 2022 8 patients were planned for LV lead pacing due to left bundle branch block, QRS duration >130 ms and LV ejection fraction below 35%. All patients (*n* = 8) scheduled for LV lead implantation had a history of atrial fibrillation and were treated *via* a minimally invasive epicardial lead implantation and concomitant LAA closure. Indications for epicardial LV lead implantation were previously unsuccessful transvenous LV-lead implantation (*n* = 6) or systemic infection with pacemaker dependency (*n* = 2). One patient with systemic infection had a complex anatomical condition of a persistent left superior vena cava. All patients gave written informed consent. All patient data were anonymized and retrospectively analyzed. All data were derived from routine in-hospital courses without follow-up. Herewith, no Institutional Review Board approval is required.

### Surgical technique

The procedure was performed using general anesthesia and a double-lumen endotracheal tube under transesophageal echo monitoring. Patient is placed in a supine position and the left side of the chest is elevated to gain access to the posterior axillary line.

### Thoracoscopic approach

**Figure 1:**
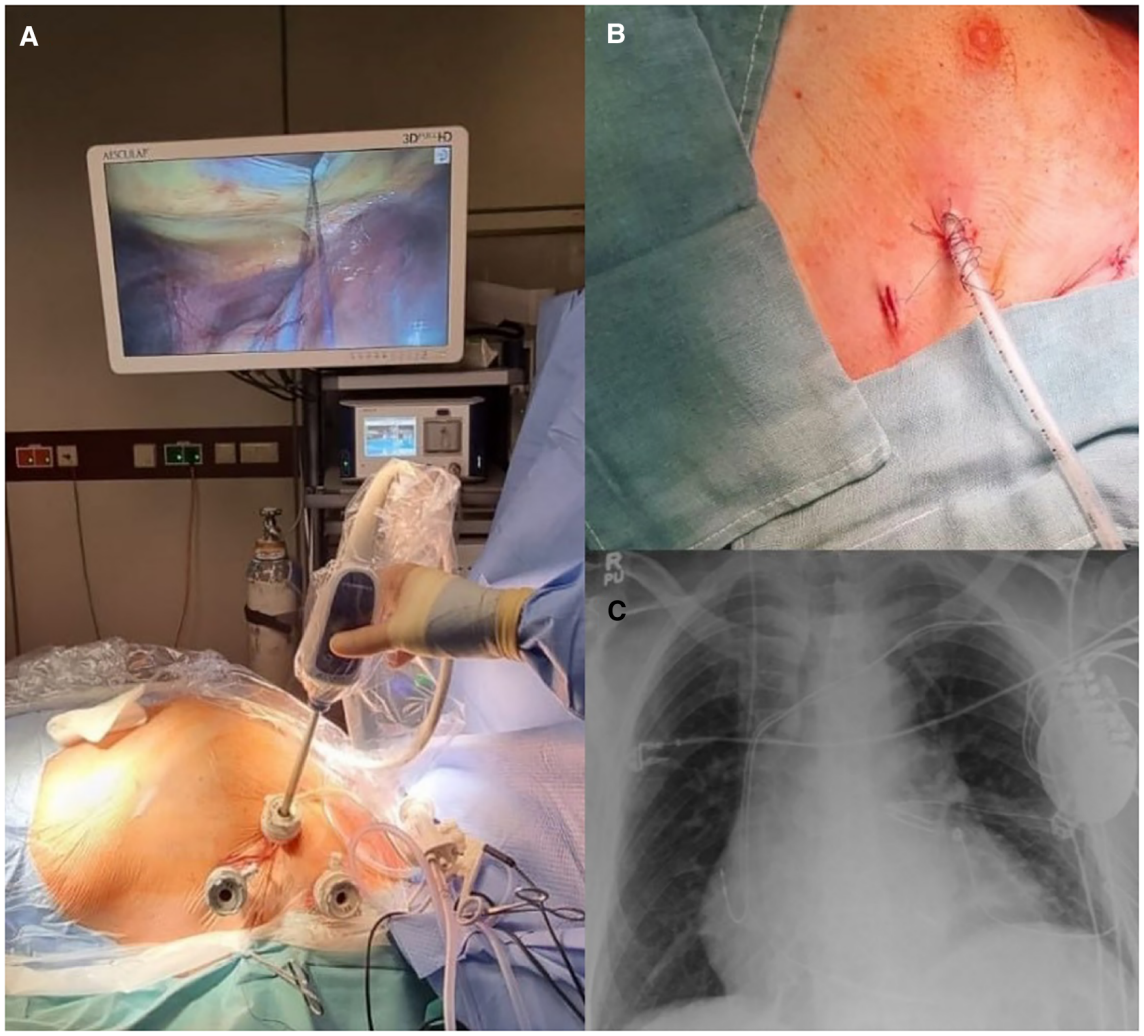
(**A**) Intraoperative picture showing the three-port access and the 3D videoscopic guidance. (**B**) Postoperative state after wound closure. (**C**) Postoperative chest x-ray showing the CRT-D device with the epicardial LV lead and the AtriClip PRO2® device.

**Figure 2:**
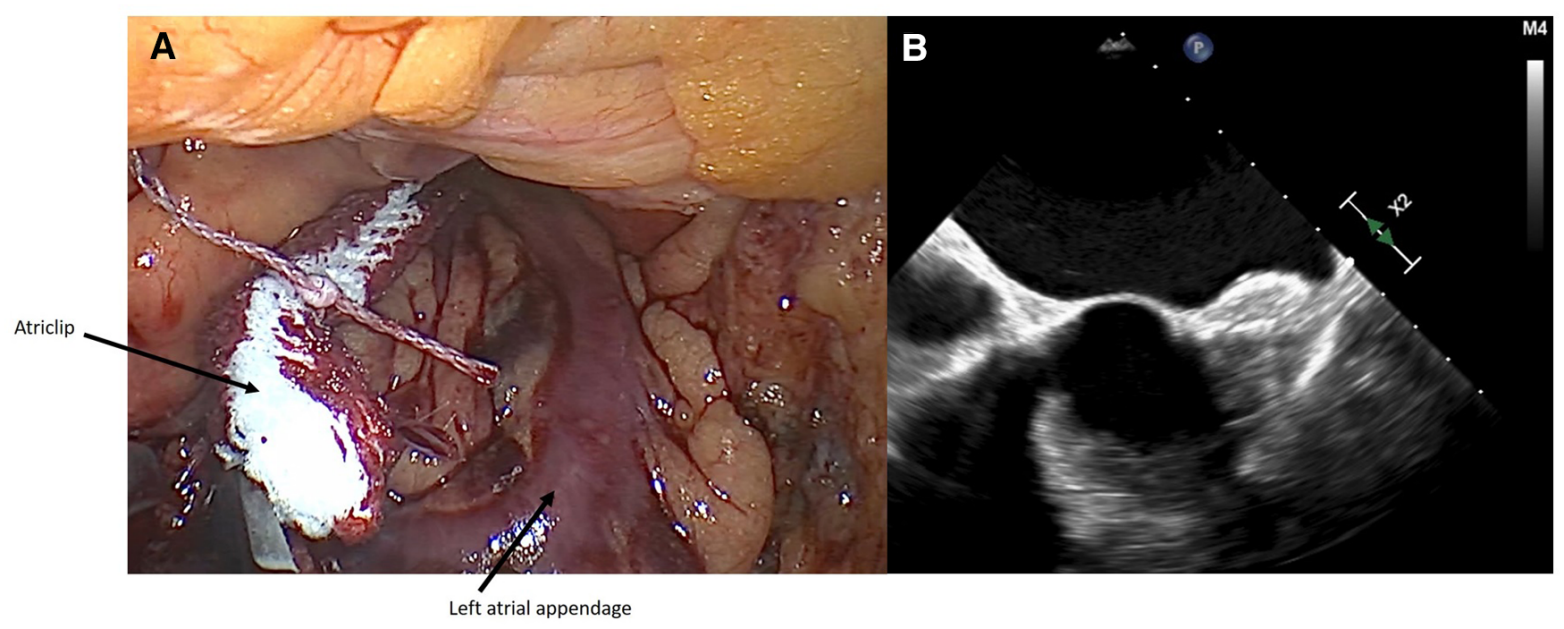
(**A**) intraoperative closure of LAA with AtriClip PRO2® device. (**B**) Intraoperative transesophageal echocardiography showing successful closure of LAA.

Totally thoracoscopic access was achieved through three-ports (Figure 1A). One 10 mm camera-port was introduced in the 6th intercostal space in the anterior axillary line and 12 mm working ports were introduced in the posterior axillary line in the 4th and 8th intercostal space. CO_2_ Insufflation was used during the procedure. Using 3D vision endoscopic guidance, the pericardium is opened 3 cm posterior to the phrenic nerve. An AtriClip PRO2® device (Atricure, West Chester, OH), which can be deployed *via* a 12 mm working port, was placed at the base of the left atrial appendage. Deployment of the AtriClip was guided by live transesophageal echocardiography in order to achieve complete LAA closure. After satisfactory placement, the AtriClip is released and the deployment device retrieved (Figure 2). Secondary, a Greatbatch Myopore™ screw-in lead was placed in the posterolateral LV-wall using a Greatbatch FASTAC™ delivery tool. Sensing, pacing threshold and impedance measurements are carried out. If the electrode values were satisfactory (Pacing threshold ≤1.5 V at 0.5 ms, Sensing ≥5 mV and Impedance between 300 and 1,500 *Ω*) the end of the lead was externalized, and tunneled into the pacemaker pocket. If measurements were not sufficient, an alternative lead position was used and measurements were performed again. At the end the pericardium is closed and a chest tube is placed through one of the working ports into the left pleural space. Ports are removed and the incisions are closed.

### Minimally invasive left-lateral thoracotomy

**Figure 3:**
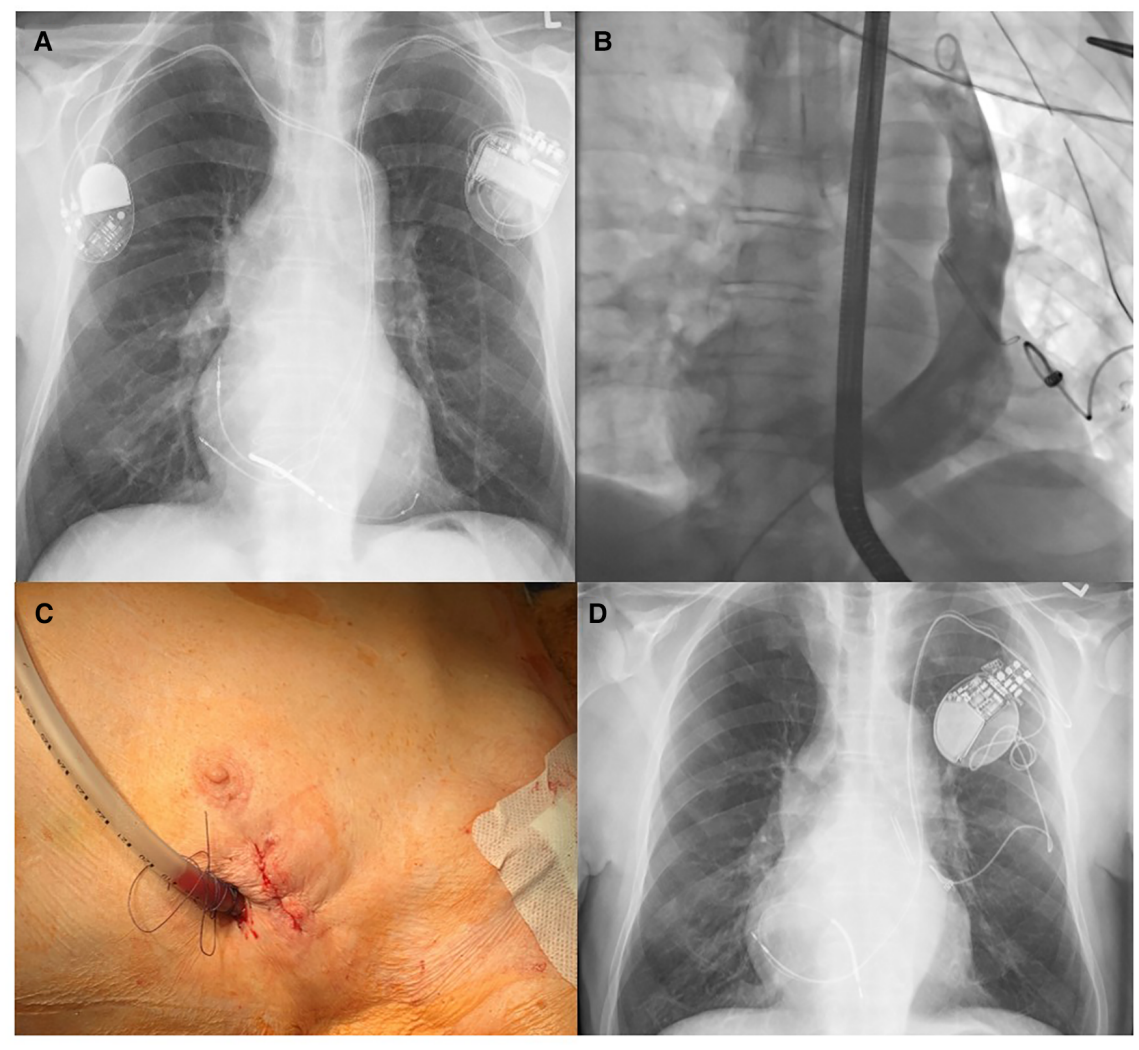
(**A**) Preoperative chest x-ray showing the left-sided CRT-D device and the right-sided pacemaker through left-sided superior vena cava (SVC). (**B**) Intraoperative venography, after extraction of all leads, displaying the left-sided SVC and the epicardial LV-lead as well as the AtriClip®. (**C**) Minimally invasive left-lateral incision in the fourth intercostal space after wound closure. (**D**) Postoperative chest x-ray showing the AtriClip® and the LV lead connected to a single-chamber pacemaker.

Minimally-invasive access was achieved *via* a 3–5 cm lateral minithoracotomy through the 4th intercostal space on the left side of the chest (Figure 3C). Next, a soft-tissue retractor is inserted. Under direct vision, the pericardium is opened 3 cm posterior to the phrenic nerve. Pericardial stay sutures are placed. An AtriClip PRO® is used to close the LAA at the base with similar confirmation by transesophageal echocardiography. Secondary, a Greatbatch Myopore^TM^ screw-in lead was placed in the posterolateral LV-wall under direct vision, without necessity of an additional delivery tool. When satisfactory lead measurements are achieved, the pericardium is closed under direct vision and a chest tube is placed.

### Laser lead extraction

Laser lead extraction was performed in two patients with systemic device related infection and pacemaker dependency as previously described (9). All procedures were performed under fluoroscopic guidance in a hybrid operating room under general anaesthesia. A transoesophageal echocardiography probe was placed to monitor for pericardial or pleural effusion. All patients were prepared for emergent sternotomy with cardiopulmonary bypass standby. Leads were dissected from the scar tissue and the sleeves were removed. Lead locking devices were placed into the lumen of the leads. Laser lead extraction was conducted using Spectranetics 16 F GlideLight (80 Hz) laser sheaths (Spectranetics Corporation, Colorado Springs, CO, USA). Laser lead extractions were performed with a single sheath technique and without outer sheaths.

### Postoperative

Patients are extubated in the operating room and transferred towards the general ward after the procedure. Chest x-ray is performed on the day of surgery. Pacemaker/ICD interrogation is performed and chest tube drain is removed on the 1st postoperative day. Anticoagulation is maintained according to CHADS_2_-VASc Score.

### Statistics

Continuous variables are expressed as mean ± standard deviation (SD) for normal distributions and median and interquartile range (IQR) for non-gaussian distributions. Categorial variables are shown as counts and percentages. Statistical analysis was performed using IBM SPSS 25.0 statistical software package (IBM, Armonk, NY, USA).

## Results

### Patient demographics

Baseline patient characteristics are outlined in Table 1. Mean patients age was 64 ± 11.2 years, 75% were male patients. Ejection fraction was severely reduced in all patients (23.8 ± 4.8%) with heart failure symptoms of NYHA class III and IV. Three patients had paroxysmal AF whereas five patients had persistent AF.

**Table 1:** Patient demographics.

	Patients (*n* = 8)
Age (years)	64 ± 11.2
Gender (Male); *n* (%)	6 (75.0)
Body Mass Index (kg/m^2^)	26.9 ± 4.5
Left ventricular ejection fraction (%)	23.8 ± 4.8
Arterial hypertension (%)	7 (87.5)
COPD (%)	2 (25.0)
Ischemic cardiomyopathy (%)	4 (50.0)
Renal failure; *n* (%)	4 (50.0)
**Atrial fibrillation; *n* (%)**
– paroxysmal	3 (37.5)
– persistent	5 (62.5)
CHADS_2_-VASc Score	4.5 ± 0.8
Catheter-based ablation (%)	7 (9)
**Anticoagulation**
– DOAK (%)	6 (75.0)
– Phenprocoumon (%)	2 (25.0)
**Antiarrhythmics**
– Betablocker	6 (75.0)
– Amiodarone	4 (50.0)
**NYHA Class; *n* (%)**
– class III	4 (50)
– class IV	4 (50)

### Perioperative data

The perioperative data is outlined in Table 2. Epicardial lead implantation was successfully performed in all patients with good pacing threshold (mean 0.8 ± 0.2 Volt at 0.5 ms) and sensing values (10.1 ± 2.3 mV). Posterolateral position of the LV lead was achieved in all patients. Furthermore, successful LAA closure was confirmed during transesophageal echocardiography in all patients. Mean procedural time with totally thoracoscopic approach (*n* = 2) was 116 min whereas the procedure time for the minimally invasive left-lateral thoracotomy approach without laser lead extraction (*n* = 4) was shorter (72 min). The procedure with additional laser lead extraction was 132 min. All patients were extubated in the OR immediately after the procedure and transferred to the general ward on the same day of surgery. No procedure related complications occurred in any of the patients.

**Table 2:** Results and complications.

	Patients (*n* = 8)
Minithoracotomy; *n* (%)	6 (75%)
Totally thoracoscopic approach	2 (25)
Concomitant laser lead extraction	2 (25)
**Type of device**
– CRT-D	7 (87.5)
– CRT-*P*	1 (12.5)
Pacing threshold (V at 0.5 ms)	0.8 ± 0.2
Sensing (mV)	10.1 ± 0.2
Impendance (*Ω*)	681 ± 248
Successful left atrial appendage closure; *n* (%)	8 (100)
Complications	0

Mean duration of in-hospital stay was 3.8 ± 1.6 days in patients without systemic device-related infection. Hospital stay of the two patients with systemic infection was prolonged due to intravenous antibiotic treatment (36 and 41 days).

## Discussion

Herewith, we highlight the possibility of epicardial LV-lead implantation and concomitant closure of the left-atrial appendage through the same totally thoracoscopic port-access- or a minimally invasive left-lateral thoracotomy.

Our described approach allows for minimally-invasive epicardial lead implantation with less surgical trauma and also enables for ideal LV-lead placement, independently from any coronary sinus anatomy. Furthermore, using this access, a posterolateral positioning of the lead can be achieved, which is essential in order to achieve a sufficient cardiac resynchronization therapy. Especially, in patients not suitable for endovascular LV lead implantation, this approach represents an excellent alternative treatment algorithm. Most recently, Burger et al. published their experiences of epicardial LV leads in 158 patients with low complication rates and excellent long-term performance with only a 1.9% lead revision rate at five years (compared to 10.2% for transvenous leads).

In addition, we have shown a treatment algorithm for patients with device endocarditis requiring lead removal, pacemaker dependency and atrial fibrillation. Systemic device related infection represents a rare but serious complication of device therapy, associated with significant morbidity and mortality (10). In systemic device related infection, extraction of all lead material is recommended as a class I indication (11). Especially, patients with pacemaker dependency and systemic infection represent a clinically challenging cohort, since there is a need for a bridging solution. Epicardial LV leads have the benefit of no foreign material in the vascular space, which helps as a bridging option until treatment of systemic infection has been accomplished. For instance, transcutaneous pacing with an externalized pacer can be used as a bridging solution with good results (12). Once the systemic infection is effectively treated, the epicardial LV lead can be easily combined with endovascular right ventricular (RV) and right atrial (RA) leads and a sufficient CRT therapy can be facilitated. Recent studies confirmed the safety of a video-assisted thoracoscopic placement of epicardial LV leads with excellent long-term outcome (6, 7).

Heart failure is often accompanied by atrial fibrillation and therefore many patients presenting for CRT-D implantation suffer from atrial fibrillation (3). A recently published prospective randomized trial, the LAAOS III Study has shown the beneficial effects of concomitant surgical LAA closure in patients with AF undergoing cardiac surgery (8). In the LAAOS III study, a significant stroke reduction has been observed during follow-up, without any evidence of procedure-related adverse events. Therefore, concomitant surgical LAA closure is strongly recommended in patients with AF undergoing any cardiac surgical procedure. This applies on the one hand to conventional surgical procedures (e.g., coronary artery bypass surgery, aortic valve replacement, or mitral valve surgery) but can also be used in other procedures like epicardial LV lead placement. Our study highlights this new approach whereas LAA closure using an epicardial clip can be performed safe and easy. Especially in patients with a high CHA_2_DS_2_-VASc Score or a history of stroke, concomitant closure of the LAA is beneficial. For instance, interventional LAA closure proved to be a therapeutic alternative in patients with high bleeding risk or contraindication for anticoagulation. In terms of stroke reduction, interventional LAA occlusion showed to be equally effective as anticoagulation therapy (13, 14). Another most recent study showed that totally thoracoscopic epicardial appendage occlusion without any antithrombotic therapy appears to be safe and effective. Branzoli et al. proposed that this strategy could be advised for stroke prevention in patients with high risk of bleeding (15).

A recent retrospective multicenter study of 175 high stroke risk patients treated by thoracoscopic stand-alone LAA closure using the AtriClip underlined the safety and efficacy of this technique. Procedural success was 99.4% and no stroke occured during a median follow-up duration of 12.5 months besides a predicted stroke rate was 4.8/100 patient-years (calculation based on median CHA_2_DS_2_-VASc Score) (16). Besides reduction of stroke risk, the AtriClip provides electrical isolation of the LAA which can be accountable for up to 27% of atrial arrhythmias (17).

Guarricini et al. reported two patients with left atrial appendage closure and concomitant LV epicardial lead implantation *via* a totally thoracoscopic approach (18). However, our study highlights different approaches to combine LV epicardial lead implantation and LAA closure in a larger patient series. Depending on the clinical scenario either totally thoracoscopic or minimally-invasive left-lateral thoracotomy can be used. This approach combines the benefits of both procedures: exact positioning of the LV lead at a posterolateral position, stroke reduction by complete occlusion of the LAA and a minimized surgical trauma with excellent cosmetic results. Combined with short procedure-times, immediate postoperative extubation and early mobilization according to the principles of enhanced recovery after surgery (19) the patients can be discharged three to four days after surgery. In addition, the totally thoracoscopic approach is a very elegant approach, which however increases the costs in comparison to a thoracotomy approach in terms of materials (FATSTAC Applicator for LV lead and Atriclip Pro2 instead of Atriclip Pro). Therefore our alternative direct vision thoracotomy approach with a 4 cm incision presents an even more cost-effective option.

## Limitation

This is a retrospective study with a small patient series. However, this is the largest reported patient series of this novel approach. Since it's a new technique, we report only in-hospital outcome and further follow-up is necessary to analyze long-term outcome and potential stroke risk reduction.

## Conclusion

Our study highlights a novel treatment approach for patients with atrial fibrillation and the necessity of epicardial LV leads due to complex anatomical conditions, device endocarditis requiring lead removal or pacemaker dependency. Placement of a posterolateral LV lead position with concomitant occlusion of the left atrial appendage *via* a minimally-invasive left-lateral thoracotomy or even a totally thoracoscopic approach is safe and feasible with superior cosmetic results and complete occlusion of the left atrial appendage.

## Data Availability

The original contributions presented in the study are included in the article/Supplementary Material, further inquiries can be directed to the corresponding author/s.
